# New clade of enigmatic early archosaurs yields insights into early pseudosuchian phylogeny and the biogeography of the archosaur radiation

**DOI:** 10.1186/1471-2148-14-128

**Published:** 2014-06-10

**Authors:** Richard J Butler, Corwin Sullivan, Martín D Ezcurra, Jun Liu, Agustina Lecuona, Roland B Sookias

**Affiliations:** 1 School of Geography, Earth and Environmental Sciences, University of Birmingham, Edgbaston, Birmingham B15 2TT, UK; 2 Key Laboratory of Vertebrate Evolution and Human Origins of Chinese Academy of Sciences, Institute of Vertebrate Paleontology and Paleoanthropology, Chinese Academy of Sciences, 100044 Beijing, China; 3 Museo Paleontológico, "Egidio Feruglio", Av. Fontana 140, Trelew, Chubut U9100GYO, Argentina

**Keywords:** Archosauria, Argentina, Biogeography, China, Gracilisuchidae, Pangaea, Phylogenetics, Suchia, Triassic

## Abstract

**Background:**

The origin and early radiation of archosaurs and closely related taxa (Archosauriformes) during the Triassic was a critical event in the evolutionary history of tetrapods. This radiation led to the dinosaur-dominated ecosystems of the Jurassic and Cretaceous, and the high present-day archosaur diversity that includes around 10,000 bird and crocodylian species. The timing and dynamics of this evolutionary radiation are currently obscured by the poorly constrained phylogenetic positions of several key early archosauriform taxa, including several species from the Middle Triassic of Argentina (*Gracilisuchus stipanicicorum*) and China (*Turfanosuchus dabanensis*, *Yonghesuchus sangbiensis*). These species act as unstable ‘wildcards’ in morphological phylogenetic analyses, reducing phylogenetic resolution.

**Results:**

We present new anatomical data for the type specimens of *G. stipanicicorum*, *T. dabanensis*, and *Y. sangbiensis*, and carry out a new morphological phylogenetic analysis of early archosaur relationships. Our results indicate that these three previously enigmatic taxa form a well-supported clade of Middle Triassic archosaurs that we refer to as Gracilisuchidae. Gracilisuchidae is placed basally within Suchia, among the pseudosuchian (crocodile-line) archosaurs. The approximately contemporaneous and morphologically similar *G. stipanicicorum* and *Y. sangbiensis* may be sister taxa within Gracilisuchidae.

**Conclusions:**

Our results provide increased resolution of the previously poorly constrained relationships of early archosaurs, with increased levels of phylogenetic support for several key early pseudosuchian clades. Moreover, they falsify previous hypotheses suggesting that *T. dabanensis* and *Y. sangbiensis* are not members of the archosaur crown group. The recognition of Gracilisuchidae provides further support for a rapid phylogenetic diversification of crown archosaurs by the Middle Triassic. The disjunct distribution of the gracilisuchid clade in China and Argentina demonstrates that early archosaurs were distributed over much or all of Pangaea although they may have initially been relatively rare members of faunal assemblages.

## Background

The origin and diversification of archosaurs during the Triassic (252.6–201.3 Ma) was a critical event in the evolutionary history of tetrapods [1-4]. This radiation took place in the aftermath of the Permo-Triassic mass extinction, the largest extinction in the history of life (e.g. [5]), and gave rise to the major clades – dinosaurs, pterosaurs and crocodylomorphs – that dominated a diverse range of niches in terrestrial ecosystems for the subsequent 135 million years of the Mesozoic (Jurassic and Cretaceous). Archosaurs remain highly diverse in present-day ecosystems, in the form of around 10,000 living species of birds and crocodylians (e.g. [6]). The radiation of archosaurs and closely related taxa (together forming the clade Archosauriformes) is therefore critical to understanding the origin and evolution of both typical Mesozoic terrestrial vertebrate faunas and the present-day biota. Although new data and analyses have led to substantial recent advances in research into early archosaur phylogenetics and diversity (e.g. [2,3,7]), understanding of the radiation remains obscured by the poorly constrained phylogenetic positions of several key early archosauriform taxa.

Among the most enigmatic of these taxa are the small-bodied carnivores *Gracilisuchus stipanicicorum*, known from multiple relatively complete and well-preserved individuals from the late Middle Triassic of Argentina [8-10], *Turfanosuchus dabanensis*, known from a single well-preserved skull and partial postcranial skeleton from the early Middle Triassic of China [11,12], and *Yonghesuchus sangbiensis*, known from two partial skulls and fragmentary postcranial remains from the late Middle Triassic of China [13]. *Gracilisuchus stipanicicorum* has been assigned to several phylogenetic positions within the crocodylian stem-lineage (Pseudosuchia), including as an ornithosuchid [8], as a sister taxon to phytosaurs [14], or deeply nested within suchian pseudosuchians as a close relative of crocodylomorphs and/or “rauisuchians” [7,15-17]. Similarly, *T. dabanensis* has been considered a euparkeriid archosauriform [11], an archosauriform close to but outside Archosauria [18], a non-pseudosuchian archosauriform of uncertain affinities [12], a very early pseudosuchian [19], or a “rauisuchiform” pseudosuchian [15], whereas *Y. sangbiensis* has been identified as a non-archosaurian archosauriform [13] that may represent the sister taxon to Archosauria [18]. The most detailed and extensive revision of early archosaur phylogeny recovered both *G. stipanicicorum* and *T. dabanensis* close to the base of Suchia [2], but both taxa were highly unstable and acted as phylogenetic ‘wildcards’, obscuring early pseudosuchian relationships [2,20]. Furthermore, this analysis did not include *Y. sangbiensis*. Determining the affinities of all three enigmatic taxa is one of the key challenges remaining in understanding early archosaur phylogeny, and thus in understanding the broader dynamics and palaeobiogeography of this evolutionary radiation.

Here, we provide new anatomical and phylogenetic evidence that *G. stipanicicorum*, *T. dabanensis*, and *Y. sangbiensis* form a previously unrecognised Middle Triassic clade of early pseudosuchians that we group together under the family name Gracilisuchidae. Gracilisuchids were among the first archosaur clades to appear in the aftermath of the Permo-Triassic extinction, and achieved a broad palaeogeographic range. This discovery provides critical clarification of the phylogenetic relationships of species involved in the early archosaur radiation.

### Institutional abbreviations

IVPP, Institute of Vertebrate Paleontology and Paleoanthropology, Beijing, China; MCZ, Museum of Comparative Zoology, Harvard University, Cambridge, USA; PULR, Museo de Paleontología, Universidad Nacional de La Rioja, La Rioja, Argentina; PVL, Paleontología de Vertebrados, Instituto Miguel Lillo, Universidad Nacional de Tucumán, Tucumán, Argentina; UFRGS, Instituto de Geociências, Universidade Federal do Rio Grande do Sul, Porto Alegre, Brazil.

## Methods

We added scorings for *Yonghesuchus sangbiensis*, based upon our original observations of the holotype of this taxon (IVPP V 12378), to the morphological cladistic data set of Nesbitt [2]. We also added a new character to the data set, resulting in a modified data matrix composed of 413 characters and 78 taxa (following the a priori pruning of the following operational taxonomic units [OTUs] that were also excluded by Nesbitt [2]: *Archosaurus rossicus*, *Prestosuchus chiniquensis*, UFRGS 0156 T, UFRGS 0152 T, *Lewisuchus admixtus* and *Pseudolagosuchus major*). In addition, several scorings for *Gracilisuchus stipanicicorum* and *Turfanosuchus dabanensis* were modified based upon new direct observations and interpretations of the relevant specimens. Additional file 1 provides details of and justifications for all rescorings and Additional file 2 is the data matrix. This data matrix is also available at TreeBASE (http://purl.org/phylo/treebase/phylows/study/TB2:S15917).

The data matrix was analysed under equally weighted parsimony using TNT 1.1 [21]. A heuristic search with 100 replicates of Wagner trees (with random addition sequence) followed by TBR branch-swapping (holding 10 trees per replicate) was performed. The best trees obtained from the replicates were subjected to a final round of TBR branch swapping. Zero length branches in any of the recovered MPTs were collapsed (rule 1 of Coddington & Scharff [22]). Characters 32, 52, 75, 121, 137, 139, 156, 168, 188, 223, 247, 258, 269, 271, 291, 297, 328, 356 and 399 were treated as additive (ordered) following Nesbitt [2], as was the new character 413. Decay indices (=Bremer support values) were calculated and a bootstrap resampling analysis, using 10,000 pseudoreplicates, was performed reporting both absolute and GC (i.e. difference between the frequencies of recovery in pseudoreplicates of the original group and the most frequently recovered contradictory group) frequencies. Templeton tests (T-t) were conducted using PAUP* 4.0 [23] to determine the significance of alternative phylogenetic topologies, with a 5% threshold for significance (p-value ≤ 0.05 = significantly less parsimonious [S]; p-value > 0.05 = non-significant [NS]).

Nesbitt & Butler [20] included the enigmatic archosaurs *Erpetosuchus granti* and *Parringtonia gracilis* within the data matrix of Nesbitt [2]. They recovered *E. granti* and *P. gracilis* within a monophyletic Erpetosuchidae; however, this clade acted as a wildcard taxon that substantially reduced phylogenetic consensus within Archosauria. Erpetosuchidae was recovered by Nesbitt & Butler [20] within a major polytomy that also included Avemetatarsalia, Ornithosuchidae, Aetosauria + *Revueltosaurus*, *Ticinosuchus* + Paracrocodylomorpha, *G. stipanicicorum* and *T. dabanensis*. It is noteworthy that Erpetosuchidae was found as the sister taxon of *T. dabanensis* in some of the most parsimonious trees recovered by Nesbitt & Butler [20]. As a result, we conducted a second analysis where we added *E. granti* and *P. gracilis* to the data matrix. Character scorings for the two erpetosuchids follow Nesbitt & Butler [20], with the exception of the scorings for the new character added here. This data matrix was therefore composed of 413 characters and 80 taxa, and was analysed under the same search criteria described above. This modified data matrix is supplied as Additional file 3, and is also available at TreeBASE as submission S15917 (http://iczn.org/code).

There is some disagreement over the identification and interpretation of the element described as the astragalus of *T. dabanensis* by Wu & Russell [12], with Nesbitt [2] scoring all astragalus characters as uncertain for *T. dabanensis*. To reflect this uncertainty, we reran both of the analyses described above, but rescored all astragalus characters (characters 354–370) as uncertain for *T. dabanensis*.

## Results

### Review of gracilisuchids: new anatomical information and synapomorphies

#### Gracilisuchus

**Figure 1:**
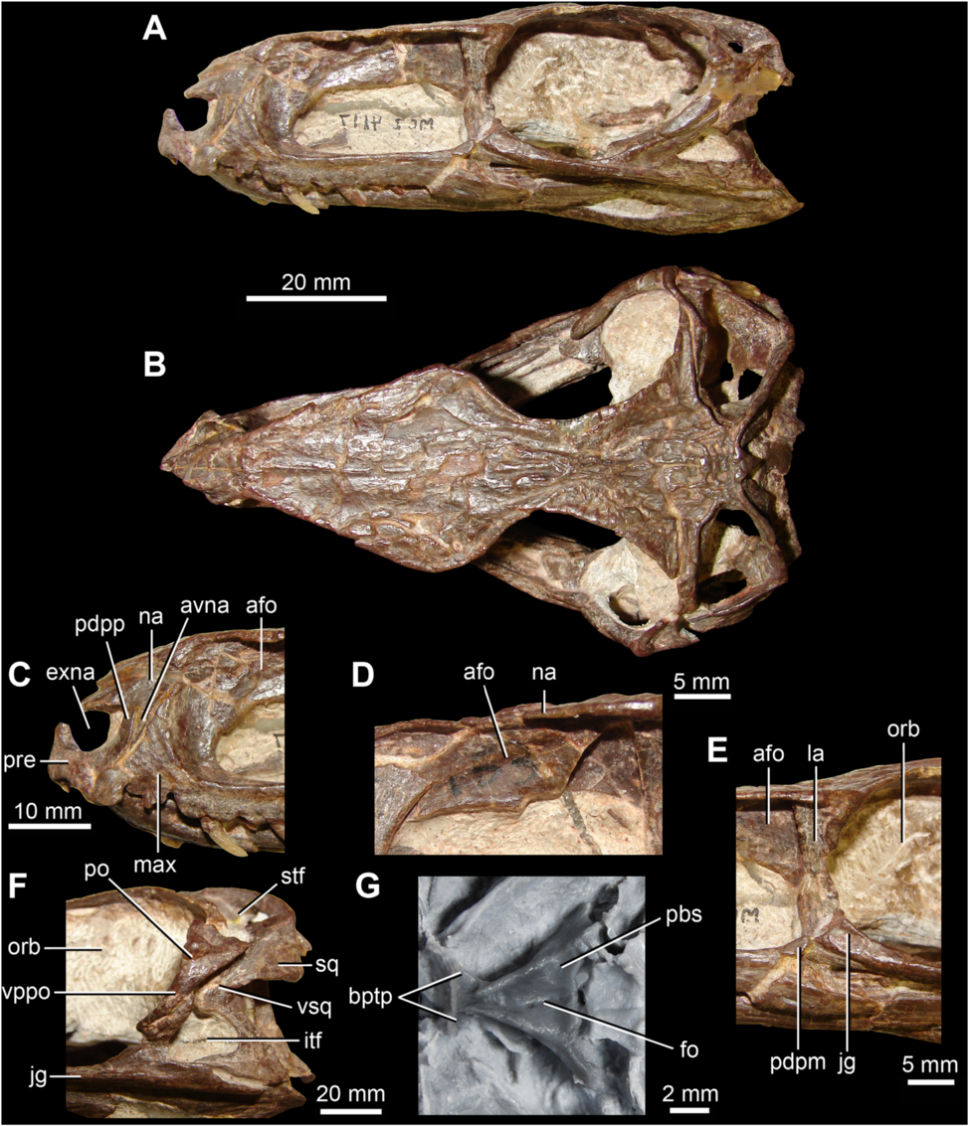
**Anatomy of *Gracilisuchus stipanicicorum *Romer **[8]**. A**. Skull in right lateral view (reversed). **B**. Skull in dorsal view. **C**. Close-up of the right premaxilla and anterior ends of right maxilla and nasal in lateral view (reversed). **D**. Close-up of the left antorbital fossa above the antorbital fenestra in lateral view. **E**. Posterodorsal process of the posterior end of the right maxilla in lateral view (reversed). **F**. Left infratemporal region in lateral view. **G**. Braincase and posterior end of the palate in ventral view. Abbreviations: afo, antorbital fossa; avna, anteroventral process of the nasal; bptp, basipterygoid process; exna, external naris; itf, infratemporal fenestra; jg, jugal; la, lacrimal; max, maxilla; na, nasal; orb, orbit; pbs, parabasisphenoid; pdpm, posterodorsal process of the posterior end of the maxilla; pdpp, posterodorsal process of the premaxilla; po, postorbital; pre, premaxilla; stf, supratemporal fenestra; sq, squamosal; vppo, ventral process of the postorbital; vsq, ventral process of the squamosal. **A-F**. MCZ 4117. **G**. cast of PULR 08.

*Gracilisuchus stipanicicorum* is known from at least six specimens (PULR 08, holotype; MCZ 4116A, 4117, 4118; PVL 4597, 4612) from the Chañares Formation of La Rioja Province, Argentina (Figure 1; [8-10]). Between these six specimens, the majority of the cranial and postcranial osteology of *G. stipanicicorum* is known. The Chañares Formation is generally considered to be of Ladinian to early Carnian age [24,25]. Because the anatomy of *G. stipanicicorum* has recently been comprehensively revised [10], and is in the process of publication by one the authors (AL), we do not provide new detailed descriptive comments here. Nevertheless, we highlight some new observations on the anatomy of *G. stipanicicorum* below in drawing comparisons to *Turfanosuchus dabanensis* and *Yonghesuchus sangbiensis*.

#### Turfanosuchus

**Figure 2:**
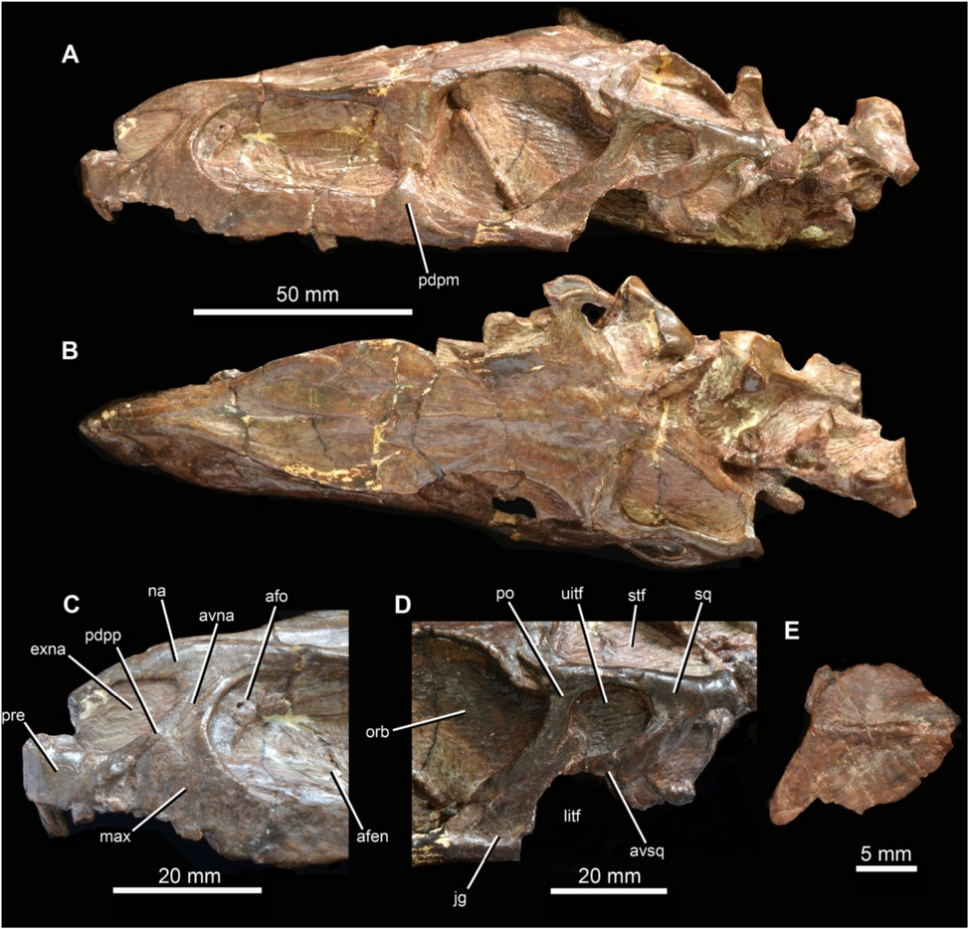
**Anatomy of the holotype skull (IVPP V3237) of *Turfanosuchus dabanensis *Young **[11]**. A**. Skull in left lateral view. **B**. Skull in dorsal view. **C**. Close-up of the left premaxilla and anterior ends of left maxilla and nasal in lateral view. **D**. Left infratemporal region in lateral view. **E**. Isolated osteoderm in dorsal view. Abbreviations: afen, antorbital fenestra; afo, antorbital fossa; avna, anteroventral process of the nasal; avsq, anteroventral process of the squamosal; exna, external naris; jg, jugal; litf, ‘lower’ subdivision of the infratemporal fenestra; max, maxilla; na, nasal; orb, orbit; pdpm, posterodorsal process of the posterior end of the maxilla; pdpp, posterodorsal process of the premaxilla; po, postorbital; pre, premaxilla; sq, squamosal; stf, supratemporal fenestra; uitf, ‘upper’ subdivision of the infratemporal fenestra.

*Turfanosuchus dabanensis* is known from a single almost complete skull and partial postcranial skeleton (IVPP V3237) from the Karamayi Formation (=Kelamayi Formation) of Xinjiang Autonomous Region, China (Figure 2; [11,12]). The presence of the dicynodont *Parakannemeyeria* in the Karamayi Formation has been used as evidence for an early Middle Triassic (Anisian) age for this rock unit (e.g. [26]). The Karamayi Formation is considered a lateral equivalent of the Ermaying Formation of Shanxi Province, which directly underlies the Tongchuan Formation that has yielded *Yonghesuchus sangbiensis* (see below). *Turfanosuchus dabanensis* was redescribed in detail by Wu & Russell [12] and its phylogenetic position discussed by Nesbitt [2]. Although a nominal second species of *Turfanosuchus*, “*T.*” *shageduensis*, has been described [27], the referral of this species to *Turfanosuchus* has not been upheld by subsequent research [28,29]. The diagnosis provided by Wu & Russell [12] for *T. dabanensis* requires modification (see below) because the first three characters listed by those authors are also present in *Y. sangbiensis*. Here, we note only a few anatomical features of *T. dabanensis* that supplement the description of Wu & Russell [12], and we provide further comparisons to *Y. sangbiensis* and *G. stipanicicorum* below.

**Figure 3:**
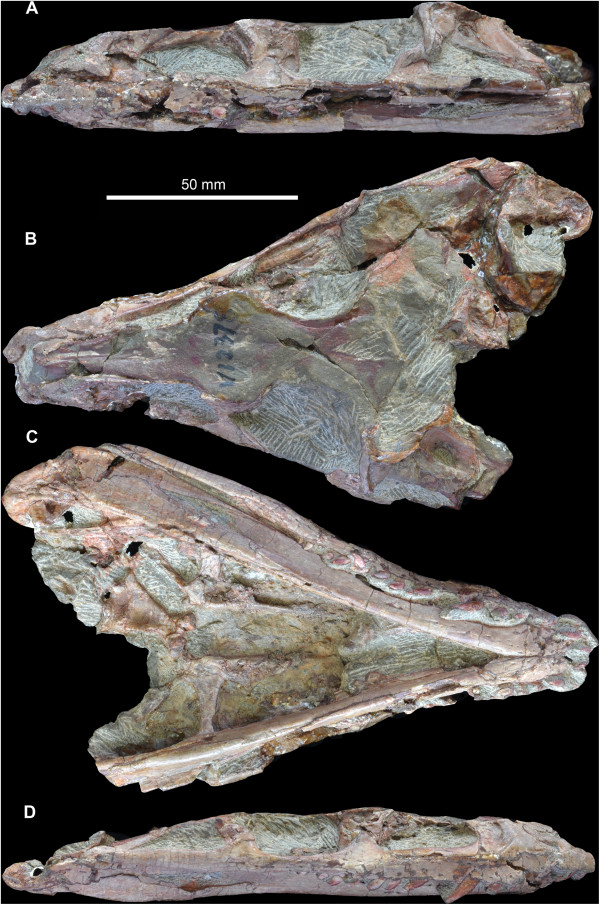
**Holotype skull (IVPP V 12378) of *Yonghesuchus sangbiensis *Wu, Liu & Li **[13]**. A**. Left lateral view. **B**. Dorsal view. **C**. Ventral view. **D**. Right lateral view.

**Figure 4:**
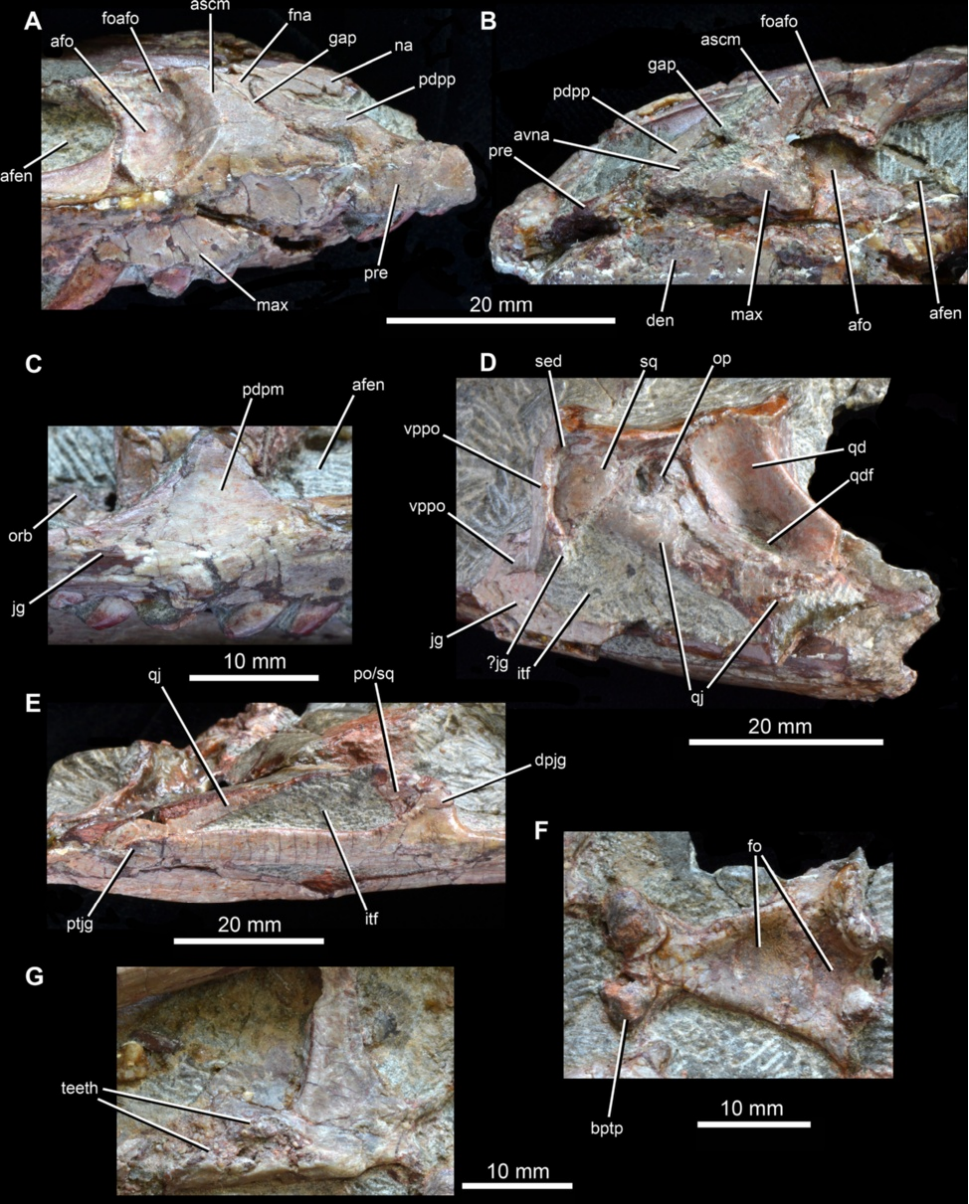
**Anatomy of the holotype skull (IVPP V 12378) of *Yonghesuchus sangbiensis *Wu, Liu & Li **[13]**. A**. Close-up of the right premaxilla and anterior end of right maxilla in lateral view. **B**. Close-up of the left premaxilla and anterior end of left maxilla in lateral view. **C**. Posterodorsal process of the posterior end of the right maxilla in lateral view. **D**. Left infratemporal region in lateral view. **E**. Right infratemporal region in lateral view. **F**. Parabasisphenoid in ventral view. **G**. Left pterygoid in ventral view. Abbreviations: afen, antorbital fenestra; afo, antorbital fossa; ascm, ascending process of the maxilla; avna, anteroventral process of the nasal; bptp, basipterygoid process; den, dentary; dpjg, dorsal process of the jugal; en, dentary; fna, facet for the nasal; fo, fossae; foafo, accessory fossa within antorbital fossa; gap, gap between the posterodorsal process of the maxilla and the anterior margin of the maxilla into which the anteroventral process of the nasal would have fitted; itf, infratemporal fenestra; jg, jugal; max, maxilla; na, nasal; op, opening between the squamosal, quadrate and quadratojugal; orb, orbit; pdpm, posterodorsal process of the posterior end of the maxilla; pdpp, posterodorsal process of the premaxilla; po/sq, fragments of the postorbital and/or squamosal; pre, premaxilla; ptjg, posterior termination of the jugal; qd, quadrate; qdf, quadrate foramen; qj, quadratojugal; sed, sediment marking a small gap between the postorbital and the squamosal; sq, squamosal; vppo, ventral process of the postorbital.

Wu & Russell [12] did not discuss the fact that the ventral end of the squamosal gives rise to a tapering anteroventral process (sensu Nesbitt [2]: character 52) that contacts or nearly contacts the dorsal process of the jugal (Figure 2D: avsq). The distal tip of the anteroventral process approaches the jugal very closely on the left side of the skull, and there is little evidence that the squamosal has been distorted or substantially displaced: the articulation of the left squamosal with the left postorbital appears to be essentially undisturbed. The ventral process of the squamosal on the right side is broken ventrally, and is therefore unnaturally shorter than would have been the case in life. Unfortunately, the quadratojugal is poorly preserved on both sides of the skull, and the nature of its articulation with the squamosal is unclear. However, we suggest that contact, or near contact, between the anteroventral process of the squamosal and the jugal probably subdivided the infratemporal fenestra into upper and lower openings, as also occurs in rauisuchids (Nesbitt [2]: character 52). The upper opening (Figure 2D: uitf) would have been bordered ventrally and posteriorly by the squamosal and anteriorly and dorsally by the ventral and posterior processes of the postorbital. Contact between the squamosal and the jugal is also present in *Y. sangbiensis* (Figures 3 and 4D; see below) and *G. stipanicicorum* (Figure 1), but in these taxa there is no upper subdivision of the infratemporal fenestra. Instead, the squamosal and postorbital have a long mutual contact, forming a wide imperforate bar between the infratemporal and supratemporal fenestra. A similar condition is also present in at least some aetosaurs (e.g. [30]). The possibility that the condition in *Y. sangbiensis* and *G. stipanicicorum* is homologous to that in *T. dabanensis* is discussed below.

Three osteoderms are present in the type specimen of *T. dabanensis*, rather than one as described by Wu & Russell [12]. One of the previously undescribed osteoderms has partially intact anterior, posterior and lateral margins (Figure 2E), and these demonstrate that the osteoderms were anteroposteriorly short with an approximately square outline. This osteoderm is anteroposteriorly shorter than individual vertebrae from the same skeleton, suggesting that more than one osteoderm would have been present per vertebra. The osteoderms are slightly asymmetrical, with a longitudinal ridge that is offset (probably laterally) from the midline, and are distinctly ventrally flexed to form an approximate L-shape in transverse section. Similar osteoderms, also with a distinct, laterally offset flexure, are present in *G. stipanicicorum* (Nesbitt [2]: character 404). Unfortunately, no osteoderms are currently known for *Y. sangbiensis*, but the presence of presacral paramedian osteoderms with a distinct ventral bend near the lateral edge optimises as a synapomorphy of Gracilisuchidae in our phylogenetic analysis.

#### Yonghesuchus

*Yonghesuchus sangbiensis* is known from Member II of the Tongchuan Formation of Shanxi Province, China, on the basis of the incomplete holotype skull (IVPP V 12378; Figures 3 and 4), and a poorly preserved paratype skull with associated cervical vertebrae (IVPP V 12379). Member II of the Tongchuan Formation has been radioisotopically dated at 238.6 ± 2.6 Ma and 234.6 ± 6.5 Ma using SHRIMP U-Pb analyses [31]. This suggests a Ladinian to Carnian age for the Tongchuan Formation, making *Y. sangbiensis* approximately contemporaneous with *Gracilisuchus stipanicicorum*[24,25].

*Yonghesuchus sangbiensis* was considered a non-archosaurian archosauriform by Wu et al. [13] and recovered as the sister taxon of Archosauria by Dilkes & Sues [18]. The non-archosaurian position of *Y. sangbiensis* in these previous studies was based upon the retention of palatal teeth on the palatal ramus of the pterygoid, given that absence of palatal teeth is the nearly universal condition in crown archosaurs and optimises as a synapomorphy of Archosauria + Phytosauria in the analysis of Nesbitt [2]. However, as discussed by Nesbitt [2], teeth are present on the palatal ramus of the pterygoid in several early dinosauriforms (e.g. *Lewisuchus admixtus*: PULR 01; *Eoraptor lunensis*: [32]; *Eodromaeus murphi*: [33]) and in *Turfanosuchus dabanensis*, which was well supported as a pseudosuchian archosaur by his phylogenetic analysis. Thus the evidence that *Y. sangbiensis* falls outside the crown group is weak.

The holotype skull of *Y. sangbiensis* (IVPP V 12378) has been crushed dorsoventrally (Figure 3), most of the skull roof has been eroded away, and many of the remaining bone surfaces are fractured or poorly preserved. As a result, interpretation of several areas of the skull is problematic. The original description of *Y. sangbiensis* by Wu et al. [13] was accurate in most respects, and those authors correctly documented several autapomorphic features that support the validity of the species. However, one of the autapomorphies that they identified (their autapomorphy 4: “posterior two thirds of descending process of postorbital broadly and deeply concave”) is considered here to reflect a misinterpretation of the temporal region of the skull. We here reinterpret several anatomical features of *Y. sangbiensis* and note other features shared with *T. dabanensis* and/or *G. stipanicicorum*.

On the right side of IVPP V 12378 (in which the premaxillary region is better preserved), there is a small gap, walled medially by a recessed area of bone, which separates the posterior margin of the tip of the posterodorsal process (= “postnarial process”, “maxillary process” or “subnarial process”) of the premaxilla from the anterior margin of the dorsal or ascending process of the maxilla (Figure 4A: gap). This gap may have been broader originally, as dorsal and posterior rotation of the premaxilla has evidently occurred post-mortem. This gap appears to be walled medially by a narrow anterior extension of the maxilla. On the left side of the skull the posterodorsal process of the premaxilla is poorly preserved, but there is a similar narrow gap between the posterodorsal process of the premaxilla and the anterior edge of the ascending process of the maxilla (Figure 4B). The ventral part of the gap contains an anteroventrally-tapering fragment of bone that likely represents the ventral tip of an anteroventral process of the nasal (Figure 4B: avna), suggesting that the gap is an articular area that accommodates this process. The nature of the articulation between the nasal and the posterodorsal process of the premaxilla in *Y. sangbiensis* would then be very similar to the condition in *T. dabanensis* (Figure 2C; [12]; [2]: character 4) and *G. stipanicicorum* (Figure 1; MCZ 4117; contra [8]) in which the nasal forms a slot to accommodate the posterodorsal process of the premaxilla and a narrow, triangular anteroventral process posterior to the slot to separate the premaxilla from the maxilla. Although the slot in the nasal is not clearly preserved in *Y. sangbiensis*, the similarities documented above lead us to score this taxon as for *T. dabanensis* and *G. stipanicicorum* for character 4 of the phylogenetic analysis, and this character state is here identified as a synapomorphy of Gracilisuchidae.

The maxilla of *Y. sangbiensis* bears a well-developed antorbital fossa that extends onto the horizontal process (= “posterior process”, or “main body”) of the maxilla below the antorbital fenestra, and continues along nearly the entire ventral margin of the fenestra. The presence of an antorbital fossa that extends onto the horizontal process was identified as a synapomorphy of crown Archosauria by Nesbitt [2], and is also present in *T. dabanensis* and *G. stipanicicorum*. Unfortunately, the palatal processes of the maxillae are not exposed in *Y. sangbiensis*, so it cannot be confirmed whether or not they contact one another along the midline as in crown archosaurs [2].

As noted by Wu et al. [13], the maxilla of *Y. sangbiensis* has a pronounced “posterodorsal process” at the posterior end of the horizontal process, posterior to the antorbital fenestra and fossa (Figure 4C: pdpm). This posterodorsal process forms a dorsoventrally deep, symmetrical, triangular projection with anterodorsal and posterodorsal margins that form steep angles to the horizontal. The process extends dorsal to the dorsal margin of the horizontal process by a distance that is almost equivalent to the height of the horizontal process of the maxilla immediately anterior to the posterodorsal process. Moreover, this process contacts the lacrimal and excludes the short anterior process of the jugal from forming part of the margin of the antorbital fenestra. A similarly well-developed process is present in *T. dabanensis* (Figure 2A), but in most archosauriforms the maxilla either tapers or maintains a nearly constant depth towards its posterior end [2]. *Gracilisuchus stipanicicorum* also has a posterodorsal process of the maxilla (Figure 1; PULR 08, PVL 4597, MCZ 4116; contra [8]). This process rises as a symmetrical triangular projection, but the anterodorsal and posterodorsal margins form less steep angles to the horizontal. Moreover, the relative height of the projection is less than in *T. dabanensis* or *Y. sangbiensis*. As such, the condition in *G. stipanicicorum* appears to be intermediate between the primitive archosauriform condition and that present in *Y. sangbiensis* and *T. dabanensis*. A similar condition to that of *G. stipanicicorum* also occurs in some, but not all, aetosaurs (e.g. [30]). The presence of a posterodorsal process of the maxilla optimises here as a synapomorphy of Gracilisuchidae.

The skull roof of IVPP V 12378 is mostly missing, but much of its outline can be reconstructed on the basis of preserved traces of bone around the margins and impressions of the ventral surfaces of some midline bones (Figure 3B). Anterior to the orbits the skull table is strongly expanded transversely at the level of the prefrontals (maximum transverse width of 30 mm as opposed to 17 mm across the orbits), similar to the condition in *T. dabanensis* and *G. stipanicicorum* (MCZ 4117), as well as in some other pseudosuchians such as ornithosuchids [34]. The skull roof tapers in transverse width anterior to this point in IVPP V 12378. It seems likely that the nasal would have formed the dorsal border of the antorbital fossa, as also occurs in *T. dabanensis*, *G. stipanicicorum* (e.g. MCZ 4117), *Aetosaurus ferratus* and several loricatan pseudosuchians and saurischian dinosaurs ([2]: character 37). Formation of the dorsal border of the antorbital fossa by the nasal optimises here as a synapomorphy of Gracilisuchidae convergently acquired in the other taxa in which it occurs [2]. Further anteriorly, dorsal to the maxillary dorsal or ascending process, the skull roof impressions suggest that the lateral margins of the nasals were thickened into ridges in *Y. sangbiensis*, as also occurs in the equivalent region in *T. dabanensis* and *G. stipanicicorum* (e.g. MCZ 4117). Unfortunately, no information is available on the outline of the frontals in *Y. sangbiensis*; however, anteriorly tapering frontals ([2]: character 43) optimise here as a synapomorphy of Gracilisuchidae, being present in both *T. dabanensis*[12] and *G. stipanicicorum* ([10]; contra [2,8]).

Examination of the infratemporal region of *Y. sangbiensis* (Figure 4D, E) in the light of comparisons to *G. stipanicicorum* suggests a somewhat different interpretation than that presented by Wu et al. [13] in the original description. The dorsal part of the posterior skull of IVPP V 12378 has been eroded away, complicating interpretation. On the left side of IVPP V 12378, the jugal-postorbital bar is preserved, but has been broken at about midheight and dorsoventrally compressed. The very tip of the ventral process of the postorbital apparently overlies the lateral surface of the jugal. A fragment of the tip of the dorsal process of the jugal that has broken away from the rest of the bone may be present posterior to the ventral termination of the postorbital (Figure 4D: ?jg).

The postorbital is incomplete dorsally, and in fact little of this bone is preserved other than the anteroposteriorly narrow ventral process and the base of the anterodorsal process. The posterior (or squamosal) process is not preserved. Immediately posterior to the dorsal two thirds of the ventral process of the postorbital, there is a broad, medially recessed area of bone that Wu et al. [13] interpreted as a fossa on the ventral process of the postorbital. However, in posterolateral view a thin line of sediment separates this recessed area from the ventral process of the postorbital (Figure 4D: sed). We interpret this recessed area of bone instead as the tip of the anterior process of the squamosal, which in *G. stipanicicorum* extends anteroventrally to contact the dorsal tip of the dorsal process of the jugal (e.g. PULR 08, MCZ 4117; Figure 1). This recessed area in IVPP V 12378 expands in anteroposterior width towards its dorsal preserved margin, where it is overhung by a ridge; a similar ridge on the squamosal that overhangs the tip of the anterior process is present in *G. stipanicicorum* (e.g. MCZ 4117; Figure 1).

The anteroventral tip of the squamosal of IVPP V 12378 is contacted posteriorly by the quadratojugal (Figure 4D). The dorsal process of the quadratojugal is directed anterodorsally in lateral view as in *G. stipanicicorum*; this does not appear to be a result of distortion. The dorsal process encloses together with the jugal and the squamosal a small lower infratemporal fenestra that has a triangular outline (Figure 4D: litf), being dorsoventrally deepest at its anterior end and sharply tapering posteriorly. The dorsal process of the left quadratojugal is damaged and part of it appears to have been slightly medially displaced, as can be inferred from a preserved impression of its original position. The quadratojugal clearly contacts the quadrate ventral to the quadrate foramen, but it is less clear whether or not there is also contact dorsal to the foramen. In the area dorsal to the quadrate foramen there is an apparent gap filled with sediment (the surface coloration of which is difficult to distinguish from that of the surrounding bone) between the anterior process of the squamosal, the quadrate, and the quadratojugal (Figure 4D: op). Whether the opening surrounded by these bones is a genuine morphological feature, or merely the result of damage to the quadrate and/or quadratojugal, cannot be determined with certainty. However, a large and apparently equivalent opening between the squamosal, quadrate and quadratojugal is present in *G. stipanicicorum* (e.g. PULR 08, MCZ 4118).The dorsal process of the right quadratojugal of IVPP V 12378 is similarly anterodorsally orientated (Figure 4E), but only its anteroventral edge is preserved. Anteriorly, the incomplete dorsal process of the jugal is contacted medially and posteriorly by some damaged fragments of bone that likely represent parts of the postorbital and squamosal. The quadratojugal also contacts these fragments, closing the small, ventrally placed infratemporal fenestra. The quadrate of IVPP V 12378 has a large quadrate foramen and a very strong ridge separating the lateral and pterygoid wings (Figure 4D).

Our reinterpretation of the temporal region of *Y. sangbiensis* reveals an unusual arrangement that is very similar to that in *G. stipanicicorum* in several respects. The squamosal forms a ventrally expanded contact with the postorbital and a more limited contact with the jugal, restricting the infratemporal fenestra to the ventral part of the lateral surface of the skull. *Yonghesuchus sangbiensis* also shares with *G. stipanicicorum* a strong ridge on the squamosal, an anterodorsally directed dorsal process of the quadratojugal, and potentially an additional opening between the squamosal, quadratojugal and quadrate. The similarities are partially captured by state 1 of character 66 of our phylogenetic analysis (“postorbital-squamosal contact continues ventrally for much or most of the ventral length of the squamosal”), which supports a sister grouping between *Y. sangbiensis* and *G. stipanicicorum*. Although the temporal region of *T. dabanensis* shows similarities to *Y. sangbiensis* and *G. stipanicicorum* such as a squamosal-jugal contact, or near contact, it also shows important differences, most notably in the probable presence of a dorsally placed opening between the squamosal and postorbital that is clearly absent in the latter two taxa. For this reason, a primary homology between the relationship of the anterior process of the squamosal to the postorbital and jugal in *T. dabanensis* versus *Y. sangbiensis* and *G. stipanicicorum* is not currently included within our phylogenetic analysis; thus *T. dabanensis* is scored as state 0 for character 66 in our analysis. However, the possibility of primary homology deserves further consideration as better preserved material is discovered.

*Yonghesuchus sangbiensis* also shows two additional noteworthy similarities to *G. stipanicicorum* that optimise as synapomorphies of a *Y. sangbiensis* + *G. stipanicicorum* clade. First, both possess a jugal that extends posteriorly beyond the level of the posterior border of the infratemporal fenestra ([2]: character 72; Figures 1 and 4E), whereas *T. dabanensis* appears to retain a plesiomorphic, proportionately shorter posterior process of the jugal, although confirmation of this character is complicated by poor preservation of this region [12]. Second, the parabasisphenoid of *Y. sangbiensis* (Figure 4F) is very similar to that of *G. stipanicicorum* (MCZ 4117; Figure 1) in being strongly elongated anteroposteriorly between the basal tubera and basipterygoid processes such that it is more than 1.5 times as long as wide ([2]: character 103). Similar elongation is not present in *T. dabanensis*[12]. Moreover, in both *Y. sangbiensis* and *G. stipanicicorum* the foramina for the internal carotid arteries are not visible in ventral view and must have entered the braincase laterally (possible synapomorphy of Archosauria following Nesbitt [2] and plesiomorphic for Gracilisuchidae), unlike in *T. dabanensis*[12].

In general, as preserved, the skull of *Y. sangbiensis* appears to be extremely similar to that of *G. stipanicicorum*. One major difference is the retention of palatal teeth on the pterygoid of *Y. sangbiensis* (Figure 4G), in contrast to the complete absence of palatal teeth in *G. stipanicicorum*[10]. The skulls of both *Y. sangbiensis* and *G. stipanicicorum* show important similarities to that of *T. dabanensis*, but *T. dabanensis* lacks some of the specialisations seen in the former two species.

### Phylogenetic analyses

**Figure 5:**
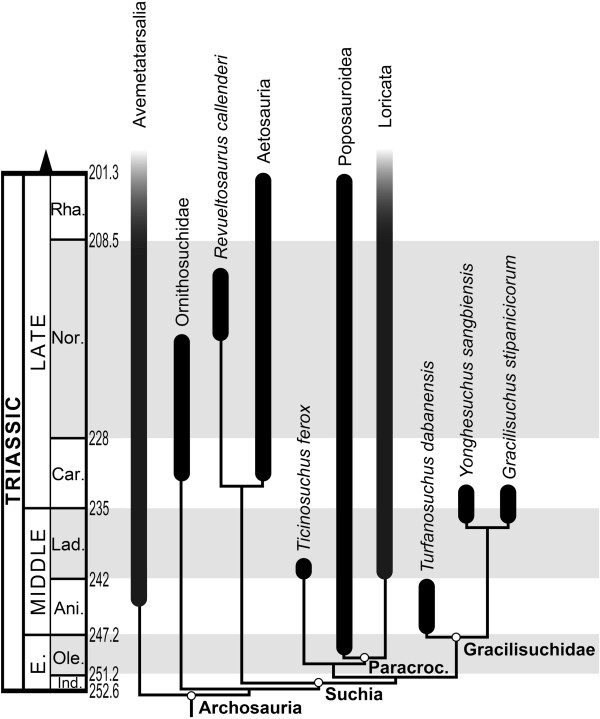
**Simplified version of the strict consensus of 90 most parsimonious trees.** Figure shows known stratigraphic ranges for major clades and stratigraphic uncertainty for gracilisuchid taxa, which are all known from point occurences.

The search recovered 90 most parsimonious trees of 1313 steps, with a consistency index (CI) of 0.3679, a retention index (RI) of 0.7706, and the best score hit in all the replications. The topology of the strict consensus tree (Figure 5; Additional file 4) is almost identical to that originally obtained by Nesbitt [2], differing only in that phylogenetic interrelationships among early suchians are completely resolved. Nesbitt [2] recovered a polytomy among *Gracilisuchus stipanicicorum*, *Turfanosuchus dabanensis*, *Revueltosaurus callenderi* + Aetosauria, and *Ticinosuchus ferox* + Paracrocodylomorpha. By contrast, in the present analysis the clade composed of *R. callenderi* + Aetosauria was recovered at the base of Suchia, and a novel clade formed by *G. stipanicicorum*, *T. dabanensis* and *Y. sangbiensis* was recovered as the sister-taxon of *T. ferox* + Paracrocodylomorpha. This novel clade is termed here Gracilisuchidae (see Systematic Palaeontology, below). The monophyly of Gracilisuchidae is supported by six unambiguous synapomorphies (see Diagnosis in the Systematic Palaeontology section, below). Within Gracilisuchidae, *G. stipanicicorum* was recovered as more closely related to *Y. sangbiensis* than to *T. dabanensis* on the basis of the following three synapomorphies: (1) postorbital-squamosal contact continues ventrally for much or most of the ventral length of the squamosal (character 66: 0 → 1); (2) jugal with a posterior termination posterior to the infratemporal fenestra (character 72: 0 → 1); (3) parabasisphenoid between basal tubera and basipterygoid processes significantly elongated, at least 1.5 times longer than wide (character 103: 0 → 1).

Gracilisuchidae is relatively strongly supported, with a Bremer support of 4 and absolute and GC bootstrap frequencies of 66% and 55%, respectively (see Additional file 4). By contrast, the recovered pattern of relationships within Gracilisuchidae is considerably less well supported, the *Y. sangbiensis* + *G. stipanicicorum* clade having a Bremer support of 1 and bootstrap frequencies below 50%. The Bremer support value for Gracilisuchidae remains unchanged following the a posteriori exclusion of either *Y. sangbiensis* or *T. dabanensis*. However, resampling values for the *G. stipanicicorum* + *Y. sangbiensis* clade increase significantly after the a posteriori exclusion of *T. dabanensis*, with new absolute and GC frequencies of 75% (difference = +32%) and 61% (difference = +42%) respectively.

The full resolution of relationships among early members of Suchia in the current analysis results in increased Bremer support values for several clades within Pseudosuchia, compared to those obtained in the original analysis of Nesbitt [2]. The clades affected are Ornithosuchidae (support increases from 8 to 13), Suchia (from 1 to 3), the *R. callenderi* + Aetosauria clade (from 9 to 10), Aetosauria (from 10 to 14), and the *T. ferox* + Paracrocodylomorpha clade (from 1 to 4). Accordingly, the results of the present analysis are consistent with those of Nesbitt [2] but provide increased support for several clades that lie close to the base of Pseudosuchia.

Analysis of suboptimal topologies shows that 10 additional steps are needed to find *Y. sangbiensis* as the sister-taxon of phytosaurs and archosaurs (relationship found by Dilkes & Sues [18]; T-t p-value = 0.0129 [S]), 13 extra steps to position *T. dabanensis* as the most basal pseudosuchian (relationship found by Ezcurra et al. [19]; T-t p-value = 0.0010 [S]) and 28 additional steps to position *G. stipanicicorum* as the most basal crocodylomorph (relationship found by several previous authors: e.g. Benton & Walker [17]; T-t p-value = 0.0002 [S]). However, only one additional step is necessary to recover a sister-taxon relationship between the Chinese taxa *T. dabanensis* and *Y. sangbiensis* (T-t p-value = 1.000 [NS]), and only three additional steps to find *G. stipanicicorum* more closely related to *T. dabanensis* than to *Y. sangbiensis* (T-t p-value = 0.2500 [NS]). In addition, only three additional steps are necessary to find a monophyletic Gracilisuchidae as the sister-taxon of *R. callenderi* + Aetosauria, and this hypothesis was not rejected by a Templeton test (T-t p-value = 0.3750 [NS]).

The analysis including erpetosuchids recovered 630 most parsimonious trees of 1330 steps, with a consistency index (CI) of 0.3632, a retention index (RI) of 0.7677, and the best score hit in 99% of replications. The topology of the strict consensus tree (Additional file 5) closely resembles that recovered by Nesbitt & Butler [20], being poorly resolved with a major polytomy at the base of Archosauria. Gracilisuchidae is recovered as monophyletic, with a Bremer support of 3 and absolute and GC bootstrap frequencies of 62% and 52%, respectively. Erpetosuchidae was found as the sister-taxon of Gracilisuchidae in some (but not all) of the most parsimonious trees, and this clade was recovered as the sister-taxon of *T. ferox* + Paracrocodylomorpha. The internal relationships among gracilisuchids are not resolved in this analysis.

Rescoring the astragalus characters as uncertain (“?”) for *T. dabanensis* did not substantially affect our results. Rerunning the main analysis (that excluding erpetosuchids) yielded 90 most parsimonious trees of 1311 steps, with the strict consensus tree showing the same topology and similar support metrics as the original analysis (Additional file 6). Rerunning the analysis that included erpetosuchids yielded 630 most parsimonious trees of 1328 steps, with the strict consensus tree showing the same topology and similar support metrics as the original analysis (Additional file 7).

### Systematic palaeontology

Archosauria Cope [35] sensu Sereno [36]

Pseudosuchia Zittel [37] sensu Nesbitt [2]

Suchia Krebs [38] sensu Nesbitt [2]

Gracilisuchidae new family

### Included taxa

*Gracilisuchus stipanicicorum* Romer [8]

*Turfanosuchus dabanensis* Young [11], and

*Yonghesuchus sangbiensis* Wu *et al*. [13].

### Phylogenetic definition

The most inclusive clade containing *Gracilisuchus stipanicicorum* Romer [8], but not *Ornithosuchus longidens* (Huxley) [39], *Aetosaurus ferratus* Fraas [40], *Poposaurus gracilis* Mehl [41], *Postosuchus kirkpatricki* Chatterjee [42], *Rutiodon carolinensis* (Emmons) [43], *Erpetosuchus granti* Newton [44], *Revueltosaurus callenderi* Hunt [45], *Crocodylus niloticus* (Laurenti) [46], or *Passer domesticus* Linnaeus [47] (new definition).

### Diagnosis

Diagnosed by the following unambiguous synapomorphies (character number in the phylogenetic analysis and reconstructed state transformation in brackets): (1) premaxilla with posterodorsal process (= “postnarial process”, “maxillary process” or “subnarial process”) that fits into slot on lateral surface of nasal (character 4: 0 → 3); (2) nasal forms part of dorsal border of antorbital fossa (character 37: 0 → 1); (3) frontal with anterior portion that tapers anteriorly along midline (character 43: 0 → 1); (4) calcaneum with dorsoventrally aligned median depression on distal end of tuber (character 375: 0 → 1); (5) presacral paramedian osteoderms with distinct longitudinal bend near lateral edge (character 404: 0 → 1); (6) maxilla with triangular posterodorsal process possessing clear dorsal apex and formed by discrete expansion of posterior end of horizontal process of maxilla (character 413: 0 → 1/2).

### Stratigraphic and geographic distribution

Known from the early Middle Triassic (Anisian) of Xinjiang, China (*Turfanosuchus dabanensis*), and the late Middle Triassic–early Late Triassic (Ladinian–early Carnian) of Shanxi, China (*Yonghesuchus sangbiensis*) and La Rioja Province, Argentina (*Gracilisuchus stipanicicorum*). The known record of gracilisuchids therefore spans the Middle Triassic and has a disjunct palaeogeographic distribution.

### Remarks

Carroll [48] listed the family Gracilisuchidae as part of a classification scheme for all fossil vertebrates, but without discussion or justification. In addition to *Gracilisuchus stipanicicorum*, Carroll [48] assigned to Gracilisuchidae the Middle Triassic taxon *Lewisuchus admixtus*, which is now considered a probable silesaurid dinosauriform [2,49]. Carroll did not provide a description or definition for Gracilisuchidae, and as such it is unavailable under Article 13.1.1 of the International Code on Zoological Nomenclature (http://iczn.org/iczn). We here provide the first valid diagnosis of Gracilisuchidae, including three genera and species, for which we provide a novel phylogenetic definition.

*Gracilisuchus stipanicicorum* Romer [8].

### Holotype

PULR 08, partially articulated skull and mandible, and partial postcranium.

### Referred material

MCZ 4116A, 4117, 4118, PVL 4597, 4612 (for details see Lecuona & Desojo [11]).

### Diagnosis

Diagnosed by the following unique combination of characters (autapomorphies marked with an asterisk): large antorbital fenestra occupying approximately 30% of anteroposterior length of skull table (measured from anterior end of premaxilla to posterior end of parietals); large antorbital fossa occupying 40% of length of skull table; presence of postfrontal and small postparietal; anterior ramus of squamosal laterally expanded; interparietal suture partially obliterated; narrow occipital portion of parietals; postzygapophyseal facet of axis horizontal, posteriorly directed, and facing ventrally*; high and vertical anterior border of axial neural spine*; presence of a ventral longitudinal median keel on axial centrum; poor development of ventral keel on cervical vertebrae; circular depression on the mid-dorsal region of the neural arch of cervical vertebrae; spine table in posterior cervical vertebrae; lack of well-defined acetabular surface on pubis; L-shaped lamina on proximal pubic apron; ischiadic symphysis proximally located*; femur longer than tibia; knob-shaped iliofibular trochanter; two paramedian osteoderms per vertebra [11].

### Type locality

Three kilometres north of the northern branch of the Chañares River and 5 km southwest of the Puerta de Talampaya, La Rioja Province, Argentina.

### Type horizon

Chañares Formation. Late Middle–early Late Triassic: Ladinian–Carnian [24,25].

*Turfanosuchus dabanensis* Young [11].

### Holotype

IVPP V3237, fairly complete skull and partial postcranium.

### Diagnosis

Characterised by the following autapomorphies: (1) dorsal or postorbital process of jugal with lateral surface strongly inset relative to lateral surface of remainder of bone, and with very broad ventral base; (2) angular excluded by surangular-dentary contact from margin of external mandibular fenestra; (3) dentary with elongate posteroventral process longer than posterodorsal process; (4) posterolateral surface of surangular highly concave (modified from Wu & Russell [12]).

### Type locality

Taoshuyuanzi, about 30 km northwest of the Turfan Basin, Xinjiang.

### Type horizon

Vertebrate Fossil Bed IV (kannemeyeriid zone), lower Kelamayi Formation. Middle Triassic: Anisian.

*Yonghesuchus sangbiensis* Wu, Liu & Li [13].

### Holotype

IVPP V 12378, incomplete skull with mandible.

### Paratype

IVPP V 12379, incomplete skull in articulation with the first seven cervical vertebrae and several cervical ribs.

### Diagnosis

Characterised by the following autapomorphies: (1) anterior ends of premaxillae tapered to form sharp point together in dorsal view; (2) accessory fossa present within antorbital fossa on dorsal part of ascending process of maxilla; (3) two fossae present on ventral surface of parabasisphenoid between basal tubera and basipterygoid processes; (4) dentary with exceptionally elongate posterodorsal process much longer than posteroventral process (modified from Wu et al. [13]).

### Type locality

North bank of Sangbi Creek, about 1.5 km southwest of Sangbi Township, Yonghe County, Shanxi province, China.

### Type horizon

Upper part of Member II of the Tongchuan Formation. Late Middle–early Late Triassic: Ladinian–Carnian [31].

## Discussion

Our phylogenetic analysis provides the first support for a gracilisuchid clade, in which we include three previously enigmatic archosauriform species from the Middle Triassic of Argentina and China: *Gracilisuchus stipanicicorum*, *Turfanosuchus dabanensis*, and *Yonghesuchus sangbiensis*. This clade is moderately well supported given that one of its members (*Y. sangbiensis*) is very incompletely known, and is resolved in a basal position within Suchia. This result provides increased resolution of the previously poorly constrained relationships of early suchians, with increased levels of support for several key early pseudosuchian clades. However, a robust resolution of the phylogenetic position of Gracilisuchidae amongst early suchians will require further work and additional discoveries. Our results contradict previous suggestions that *T. dabanensis* and *Y. sangbiensis* are non-archosaurian archosauriforms, placing both within crown Archosauria (see also Ezcurra et al. [19] and Nesbitt [2] for *T. dabanensis*).

Within Gracilisuchidae, we find a sister-group relationship between *G. stipanicicorum* and *Y. sangbiensis*. We consider this plausible given our reinterpretation of the unusual temporal region of *Y. sangbiensis* as highly similar to that of *G. stipanicicorum*, and the similar chronostratigraphic position of both taxa relative to the geologically older *T. dabanensis*. Despite this, the sister group relationship is poorly supported, perhaps due to the incomplete nature of the available material of *Y. sangbiensis*. Discoveries of more complete specimens of this taxon will be required in order to achieve a more robust understanding of relationships within Gracilisuchidae.

The oldest crown archosaur body fossils are of poposauroid pseudosuchians, particularly ctenosauriscids, the oldest records of which are clustered around the Early Triassic–Middle Triassic (Olenekian–Anisian) boundary [2,3,50]. The Manda beds of Tanzania, supported as late Anisian in age on the basis of terrestrial biochronology, represent the oldest rock unit to yield a diversity of crown archosaur clades, including a silesaurid dinosauromorph [51], an erpetosuchid [20], a poposauroid [52], a probable paracrocodylomorph [53], as well as a derived dinosauriform [54] and several undescribed species. The recognition of Gracilisuchidae, whose oldest member (*T. dabanensis*) is known from the Anisian of China, adds this clade to the roster of crown archosaur groups known from the early Middle Triassic. The occurrence of *T. dabanensis* in western China reinforces the previous evidence based on the distribution of poposauroids that early archosaurs were distributed over much or all of Pangaea from the end of the Early Triassic onwards [3], but may have initially been relatively rare members of faunal assemblages. The high diversity of early archosaur clades recovered from the Manda beds of Tanzania may reflect the late Anisian age of this formation, with the late Anisian potentially marking the point of transition from early Middle Triassic (early Anisian) vertebrate communities with only scarce crown archosaurs to late Middle Triassic (Ladinian) vertebrate communities in which crown archosaurs were diverse and relatively abundant (e.g. the Chañares Formation of Argentina).

**Figure 6:**
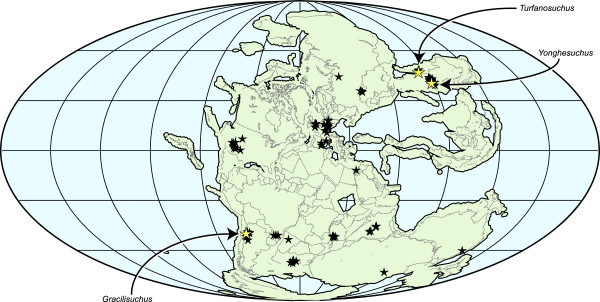
**Palaeogeographic distribution of gracilisuchids on the supercontinent Pangaea during the Middle Triassic.** Red stars indicate gracilisuchid records; black stars are other Middle Triassic records of terrestrial tetrapods. Map generated using Fossilworks tools created by Alroy [57] using maps created by Scotese [58] and data from the Paleobiology Database (http://www.paleobiodb.org). Explicit permission has been granted by John Alroy, owner of the Fossilworks site, to use this map in a CC-BY publication. Paleobiology Database data is made available under a CC-BY licence.

During the late Middle Triassic–early Late Triassic (Ladinian–early Carnian), gracilisuchids had a broad distribution (Figure 6), being present in both the Tongchuan Formation of China (*Y. sangbiensis*) and the Chañares Formation of Argentina (*G. stipanicicorum*) at approximately similar northern and southern mid-palaeolatitudes (around 40–50° N and S). Although this distribution is disjunct, Ladinian and early Carnian terrestrial vertebrate assemblages are scarce worldwide: for example, in China, *Y. sangbiensis* represents the only specifically diagnosable body fossil material yet described from this time interval. As a result, it is possible that gracilisuchids could have been widespread across Pangaea in the Ladinian and early Carnian, consistent with suggestions of relatively cosmopolitan vertebrate faunas during the Middle Triassic [55,56].

## Conclusions

Based upon new anatomical observations and a revised morphological phylogenetic dataset, we demonstrate the existence of a previously unrecognised clade of early pseudosuchian archosaurs that includes three previously enigmatic archosauriform species from the Middle Triassic of Argentina and China. We term this clade Gracilisuchidae. Our results provide increased resolution of the previously poorly constrained relationships of early archosaurs, with increased levels of phylogenetic support for several key early pseudosuchian clades. Our recognition of Gracilisuchidae provides further support for a rapid phylogenetic diversification of archosaurs by the Middle Triassic. This radiation occurred in the aftermath of the Permo-Triassic mass extinction as part of the broader recovery of post-extinction terrestrial ecosystems. Gracilisuchids occur at approximately similar northern and southern mid-palaeolatitudes (around 40–50° N and S). This disjunct distribution demonstrates that early archosaurs were distributed over much or all of Pangaea by the early Middle Triassic although they may have initially been relatively rare members of faunal assemblages.

## Availability of supporting data

The data sets supporting the results of this article are included within the article (and its files).

## Competing interests

The authors declare we have no competing interests.

## Authors’ contributions

RJB, CS and MDE collected anatomical data and carried out phylogenetic analyses. JL provided data on geological setting and JL, AL and RBS provided comparative data on other archosauriforms. RJB, CS and MDE drafted the manuscript, and RJB and MDE prepared the figures. All authors reviewed, edited and approved the final manuscript.

## Supplementary Material

Additional file 1 Notes on character scorings used in the phylogenetic dataset. Click here for file

Additional file 2 Morphological dataset used in main cladistic analysis. Click here for file

Additional file 3 Morphological dataset used in cladistic analysis incorporating Erpetosuchidae. Click here for file

Additional file 4 Strict consensus tree for the main cladistic analysis, with nodal values for Bremer support and absolute and GC bootstrap frequencies. Click here for file

Additional file 5 Strict consensus tree for the cladistic analysis incorporating Erpetosuchidae, with nodal values for Bremer support and absolute and GC bootstrap frequencies. Click here for file

Additional file 6 **Strict consensus tree for the main cladistic analysis when astragalus characters are all coded as uncertain for *Turfanosuchus dabanensis*, with nodal values for Bremer support and absolute and GC bootstrap frequencies.** Click here for file

Additional file 7 **Strict consensus tree for the cladistic analysis incorporating Erpetosuchidae when astragalus characters are all coded as uncertain for *Turfanosuchus dabanensis*, with nodal values for Bremer support and absolute and GC bootstrap frequencies.** Click here for file
