# Supplementary material for: New clade of enigmatic early archosaurs yields insights into early pseudosuchian phylogeny and the biogeography of the archosaur radiation
Source: BMC Evol Biol. 2014 Jun 10;14:128. doi: 10.1186/1471-2148-14-128 (PMC4061117; doi:10.1186/1471-2148-14-128)
Supplement: Additional file 1 — Notes on character scorings used in the phylogenetic dataset. [file 1471-2148-14-128-S1.docx]

**Supplementary material to:**

**New clade of enigmatic early archosaurs yields insights into early pseudosuchian phylogeny and the biogeography of the archosaur radiation**

**Richard J. Butler^1§^, Corwin Sullivan^2^, Martín D. Ezcurra^1^, Jun Liu^2^, Agustina Lecuona^3^, Roland B. Sookias^1^**

^1^School of Geography, Earth and Environmental Sciences, University of Birmingham, Edgbaston, Birmingham B15 2TT, UK

^2^Key Laboratory of Vertebrate Evolution and Human Origins of Chinese Academy of Sciences, Institute of Vertebrate Paleontology and Paleoanthropology, Chinese Academy of Sciences, 100044 Beijing, China

^3^Museo Paleontológico "Egidio Feruglio", Av. Fontana 140, U9100GYO Trelew, Chubut, Argentina

**Notes on character scorings used in the phylogenetic dataset**

Here we provide details on character scores that have been changed in the current dataset from those presented by Nesbitt (2011). Re-scorings for *Gracilisuchus* are based upon re-examination of the hypodigm (MCZ 4116A, 4117, 4118, PVL 4597, 4612) by AL and MDE. Additional information on some of these re-scorings are available in Lecuona (2013), available on request from AL.

Character 4. As discussed in the main text, *Yonghesuchus*, *Turfanosuchus* and *Gracilisuchus* all appear to possess state (3) of this character, with the posterodorsal process of the premaxilla fitting into a slot on the nasal. Nesbitt (2011) scored *Gracilisuchus* as possessing state (0); we here rescore *Gracilisuchus* to state (3).

Character 6. *Gracilisuchus* rescored to state (1) from state (0).

Character 12. Although the right premaxilla is slightly displaced in *Yonghesuchus*, it appears to be separated from the maxilla by a small foramen whose margins are formed by both elements (state 1). In *Turfanosuchus* there is at a slit-like, dorsoventrally elongate depression between the premaxilla and maxilla, but the depression may be floored by small recessed areas of both bones rather than penetrating between them as a foramen. We rescore *Turfanosuchus* to state (?) from state (0).

Character 15. Teeth are not preserved on the posterior half of the maxilla in *Turfanosuchus* and so we rescore this taxon to state (?) from state (0). *Gracilisuchus* rescored to state (0&1) from state (0).

Character 22. The presence of interdental plates is uncertain in both *Yonghesuchus* and *Turfanosuchus* because the specimens are preserved with the mandible adducted and so the medial surface of the tooth rows are not visible. We rescore *Turfanosuchus* to state (?) from state (0).

Character 27. We propose a modification to this character to describe whether the portion of the posterior process of the maxilla that lies below the anterior three-quarters of the antorbital fenestra, not including the posterior end of the maxilla (whether or not it lies below the fenestra), tapers posteriorly (0), is approximately constant in dorsoventral depth (1), or becomes dorsoventrally deeper posteriorly (2). We score both *Yonghesuchus* and *Turfanosuchus* as state (0), because the posterior process tapers below the antorbital fenestra in these taxa before forming a posterodorsal process the fenestra (see character 413). We also rescore this character for *Erythrosuchus* (to 2 from 0), *Chanaresuchus* (to 0&1 from 0; based on MCZ 4039 and PVL 4586, which both possess state 0, and PULR 07, which has state 1), *Euparkeria* (to 2 from 1), *Prolacerta* (to ? from 0, as this character is inapplicable given the absence of an antorbital fenestra in this taxon), and *Tropidosuchus* (to 0&1 from ? based on the holotype PVL 4601, in which the morphology appears to vary on opposite sides of the skull). The aetosaurs *Stagonolepis*, *Longosuchus* and *Aetosaurus* are all rescored to state (0) from state (2), because all have maxillae that taper posteriorly beneath the antorbital fenestra before expanding dorsally to form a discrete posterodorsal process posterior to the fenestra (see character 413).

Character 32. *Gracilisuchus* rescored to state (1) from state (?).

Character 37. Although the nasal is not preserved dorsal to the antorbital fossa in *Yonghesuchus*, the fossa appears to extend to the dorsal border of the maxilla, implying that the nasal would have entered the margin of the fossa (state 1).

Character 42. A weakly developed longitudinal ridge is present along the midline between the frontals in *Turfanosuchus* and we therefore rescore this taxon to state (1) from state (0).

Character 43. *Gracilisuchus* rescored to state (1) from state (0). *Turfanosuchus* also rescored to state (1) from state (0).

Character 45. *Turfanosuchus* rescored to state (?) from state (0) due to the incomplete preservation of the quadratojugal. *Gracilisuchus* rescored to state (1) from state (0).

Character 49. *Gracilisuchus* rescored to state (1) from state (0).

Character 55. *Gracilisuchus* rescored to state (1) from state (0).

Character 56. *Gracilisuchus* rescored to state (?) from (0) because the condition in this taxon is not clearly homologous with the ventral process of other taxa, and thus we consider the character inapplicable. We also score *Yonghesuchus* as inapplicable.

Character 58. *Gracilisuchus* rescored to state (1) from state (0).

Character 61. *Turfanosuchus* rescored to state (?) from state (0).

Character 67. *Turfanosuchus* rescored to state (1) from state (0).

Character 69. *Gracilisuchus* rescored to state (1) from state (0).

Character 75. We have modified this character because we have been unable to recognise clear differences between states (1) and (2) of Nesbitt (2013). As a result, we have combined states 1 and 2 to create a three state character that we treat as ordered/additive. Jugal, longitudinal ridge on the lateral surface of the body: absent (0); present (1); present and forms a bulbous ridge (2).

Character 84. *Turfanosuchus* rescored to state (1) from state (0). *Gracilisuchus* rescored to state (?) from state (0).

Character 85. *Turfanosuchus* rescored to state (0) from state (?).

Character 87. *Turfanosuchus* rescored to state (?) from state (0).

Character 94. *Turfanosuchus* rescored to state (0) from state (1).

Character 95. Scored as state (1 or 2) for *Yonghesuchus*, because the internal carotid clearly does not enter ventrally, but the lateral surfaces of the basisphenoid are not exposed. We score *Gracilisuchus* as state (1 or 2) because the internal carotid also clearly does not enter ventrally.

Character 101. *Gracilisuchus* rescored to state (?) from state (0).

Character 108. *Gracilisuchus* rescored to state (0&1) from state (0).

Character 111. *Gracilisuchus* rescored to state (0) from state (?).

Character 115. *Turfanosuchus* rescored to state (?) from state (0), because the floor of the endocranial cavity is not visible. *Gracilisuchus* rescored to state (0&1) from state (?).

Character 126. *Gracilisuchus* rescored to state (1) from state (?).

Character 132. *Gracilisuchus* rescored to state (0) from state (?).

Character 146. *Turfanosuchus* rescored to state (?) from state (1), because the posterior margin of the parietal is damaged and thus the presence or absence of a postparietal cannot be assessed. *Gracilisuchus* rescored to state (0&1) from state (1), based on Lecuona (2013), who identified a postparietal as present in MCZ 4117.

Character 152. *Gracilisuchus* rescored to state (0) from state (?).

Character 153. *Gracilisuchus* rescored to state (1) from state (?).

Character 163. *Gracilisuchus* rescored to state (?) from state (0).

Character 177. *Turfanosuchus* rescored to state (?) from state (1) because the vertebral column is not well enough exposed to determine whether there are intercentra.

Character 178. *Gracilisuchus* rescored to state (?) from state (0).

Character 179. *Turfanosuchus* rescored to state (0) from state (?).

Character 181. *Gracilisuchus* rescored to state (1) from state (0).

Character 182. *Turfanosuchus* rescored to state (0) from state (?). *Gracilisuchus* rescored to state (0) from state (?).

Character 190. *Turfanosuchus* rescored to (?) from (0). *Gracilisuchus* rescored to state (0) from state (?).

Character 192. *Gracilisuchus* rescored to state (?) from state (0).

Character 195. *Turfanosuchus* rescored to state (?) from state (0). *Gracilisuchus* rescored to state (?) from state (0).

Character 197. *Gracilisuchus* rescored to state (1) from state (?).

Character 198. *Turfanosuchus* rescored to state (0) from state (?).

Character 211. *Turfanosuchus* rescored to state (0) from state (?). *Gracilisuchus* rescored to state (0) from state (?).

Character 212. *Turfanosuchus* rescored to state (0) from state (?). *Gracilisuchus* rescored to state (?) from state (0).

Character 234. *Turfanosuchus* rescored to state (?) from state (0).

Character 278. *Gracilisuchus* rescored to state (0) from state (1).

Character 286. *Turfanosuchus* rescored to (?) from (0). *Gracilisuchus* rescored to state (?) from state (0).

Character 287. *Gracilisuchus* rescored to state (2) from state (0).

Character 297. *Gracilisuchus* rescored to state (?) from state (0).

Character 300. *Turfanosuchus* rescored to state (1) from state (?). *Gracilisuchus* rescored to state (0) from state (?).

Character 301. *Turfanosuchus* rescored to state (1) from state (?).

Character 305. *Gracilisuchus* rescored to state (1) from state (?).

Character 313. *Gracilisuchus* rescored to state (?) from state (0).

Character 320. *Gracilisuchus* rescored to state (1) from state (0).

Character 328. *Gracilisuchus* rescored to state (?) from state (0).

Character 330. *Turfanosuchus* rescored to state (0) from state (1).

Character 337. *Turfanosuchus* rescored to state (1) from state (?).

Character 339. *Turfanosuchus* rescored to state (?) from state (1).

Character 340. *Turfanosuchus* rescored to state (?) from state (0).

Character 341. *Turfanosuchus* rescored to state (1) from state (0).

Character 347. *Gracilisuchus* rescored to state (?) from state (0).

Character 352. *Gracilisuchus* rescored to state (1) from state (?).

Character 353. *Gracilisuchus* rescored to state (1) from state (?).

Character 354. *Turfanosuchus* rescored to state (0) from state (?).

Character 355. *Turfanosuchus* rescored to state (0) from state (?).

Character 356. *Turfanosuchus* rescored to state (0) from state (?).

Character 357. *Turfanosuchus* rescored to state (0) from state (?).

Character 361. *Turfanosuchus* rescored to state (0) from state (?).

Character 362. *Turfanosuchus* rescored to state (0) from state (?).

Character 363. *Turfanosuchus* rescored to state (1) from state (?).

Character 364. *Turfanosuchus* rescored to state (0) from state (?).

Character 365. *Turfanosuchus* rescored to state (1) from state (?).

Character 366. *Turfanosuchus* rescored to state (0) from state (?).

Character 367. *Gracilisuchus* rescored to state (1) from state (0).

Character 371. *Turfanosuchus* rescored to state (1 or 2) from state (1). *Gracilisuchus* rescored to state (2) from state (1).

Character 372. *Turfanosuchus* rescored to (0) from (1). *Gracilisuchus* rescored to state (0) from state (1).

Character 375. *Turfanosuchus* rescored to state (1) from state (?). *Gracilisuchus* rescored to state (1) from state (0).

Character 389. *Turfanosuchus* rescored to state (0) from state (?).

Character 397. *Turfanosuchus* rescored to state (0) from state (?).

Character 398. *Turfanosuchus* rescored to state (0) from state (?). *Gracilisuchus* rescored to state (0) from state (1). *Riojasuchus* rescored to state (0) from state (1), and *Ornithosuchus* rescored to state (?) from state (1).

Character 404. *Turfanosuchus* and *Gracilisuchus* rescored to state (1) from state (0).

Character 407. *Euparkeria capensis* rescored to state (1) from state (0).

Character 408. *G. stipanicicorum* rescored to (?) from (0).

Character 410. *G. stipanicicorum* rescored to (0) from (1).

Character 413. Maxilla with triangular posterodorsal process possessing clear dorsal apex and formed by discrete expansion of posterior end of horizontal process of maxilla: absent (0); present, but weakly developed, extends dorsal to the dorsal margin of the horizontal process by a distance that is less than the height of the horizontal process of the maxilla immediately anterior to the posterodorsal process (1); present and strongly developed, extends dorsal to the dorsal margin of the horizontal process by a distance that is almost equivalent to the height of the horizontal process of the maxilla immediately anterior to the posterodorsal process (2). This character is treated as additive/ordered.

**References**

Lecuona A: **Anatomía y relaciones filogenéticas de *Gracilisuchus stipanicicorum* y sus implicancias en el origen de Crocodylomorpha.** *PhD thesis.* Universidad de Buenos Aires, Facultad de Ciencias Exactas y Naturales; 2013.

Nesbitt SJ: **The early evolution of archosaurs: relationships and the origin of major clades.** *B Am Mus Nat Hist* 2011, **352:**1–292.
