# Supplementary figures and images for: New clade of enigmatic early archosaurs yields insights into early pseudosuchian phylogeny and the biogeography of the archosaur radiation
Source: BMC Evol Biol. 2014 Jun 10;14:128. doi: 10.1186/1471-2148-14-128 (PMC4061117; doi:10.1186/1471-2148-14-128)

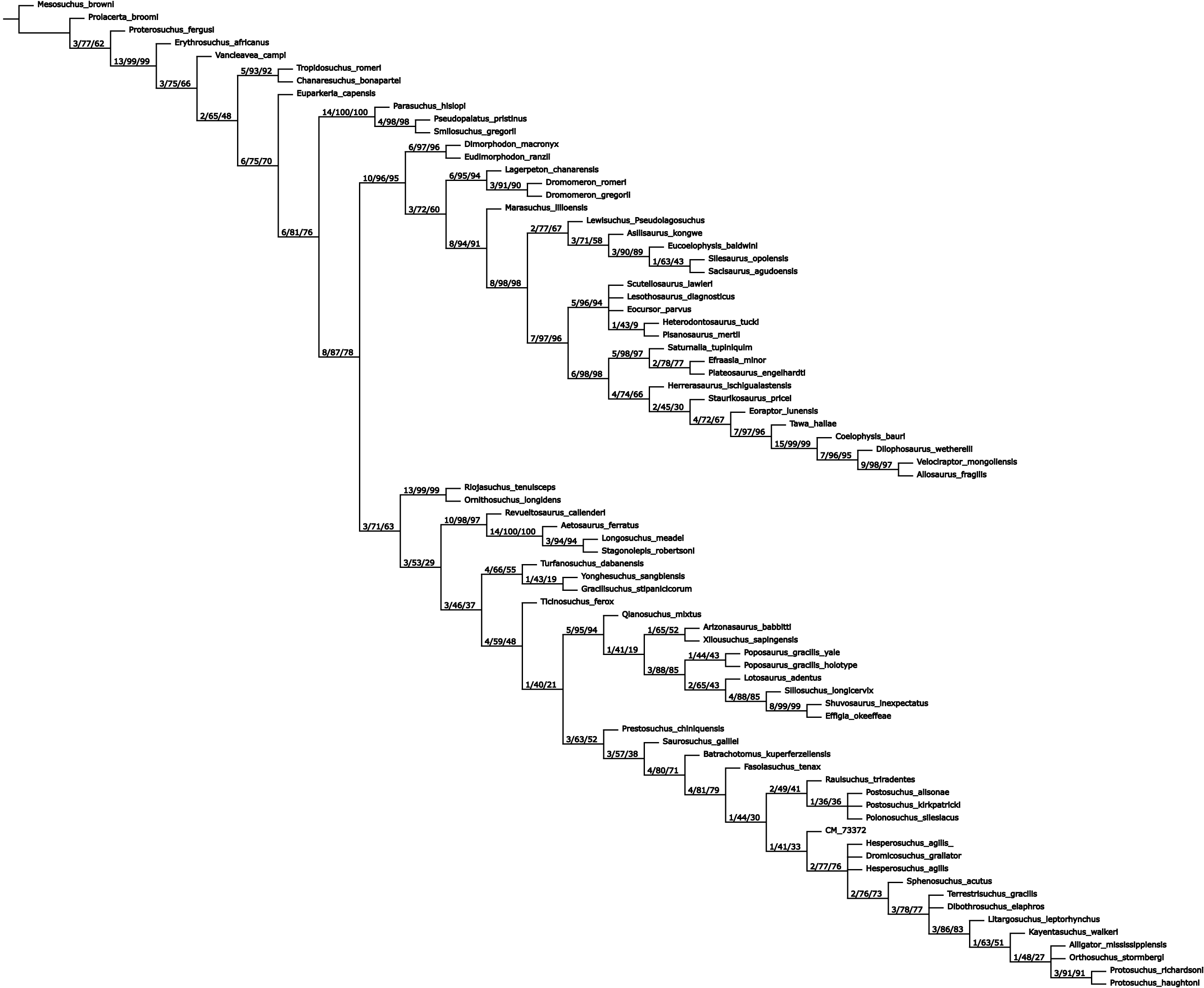

Supplement: Additional file 4 — Strict consensus tree for the main cladistic analysis, with nodal values for Bremer support and absolute and GC bootstrap frequencies. [file 1471-2148-14-128-S4.tiff]

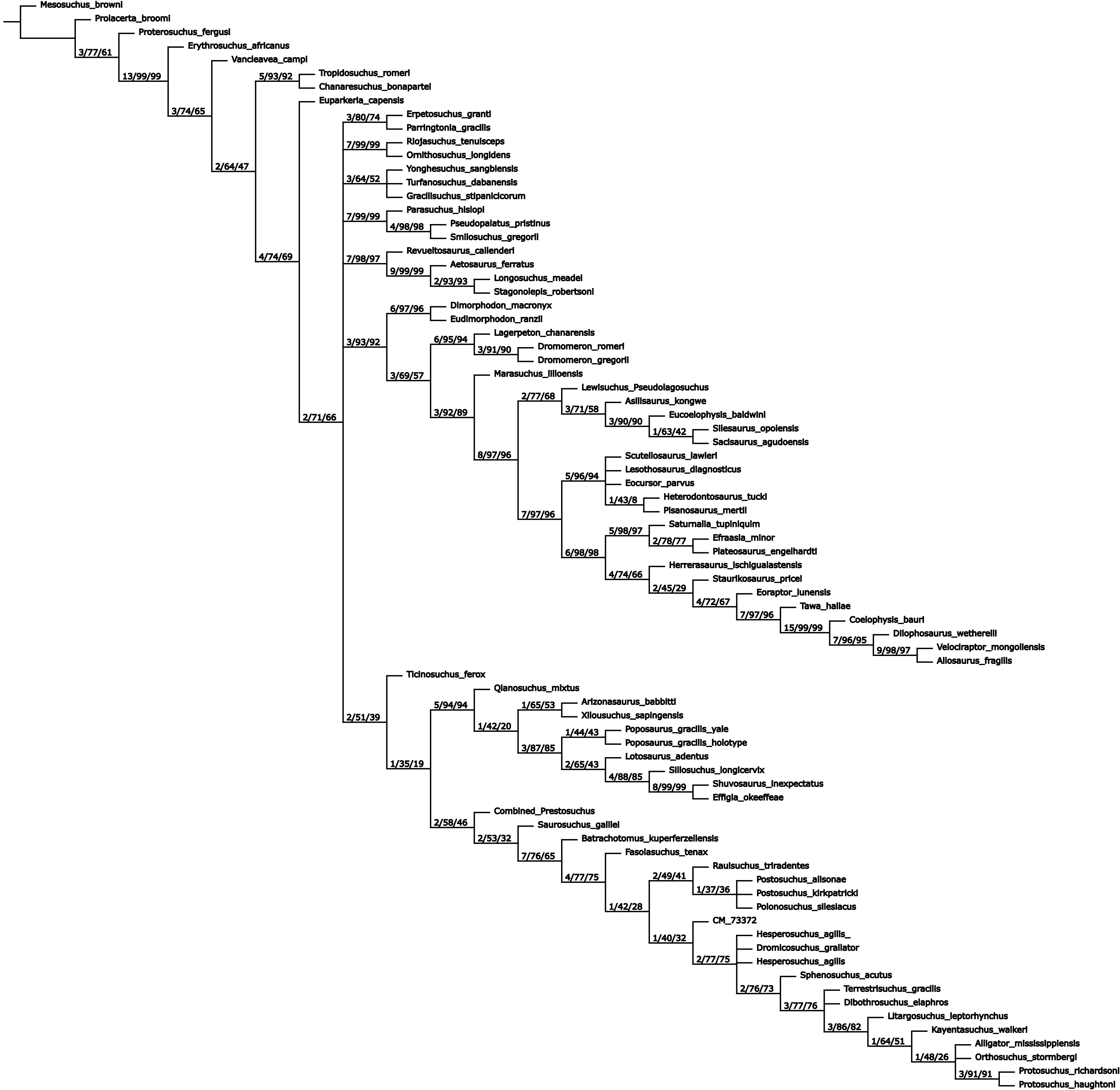

Supplement: Additional file 5 — Strict consensus tree for the cladistic analysis incorporating Erpetosuchidae, with nodal values for Bremer support and absolute and GC bootstrap frequencies. [file 1471-2148-14-128-S5.tiff]

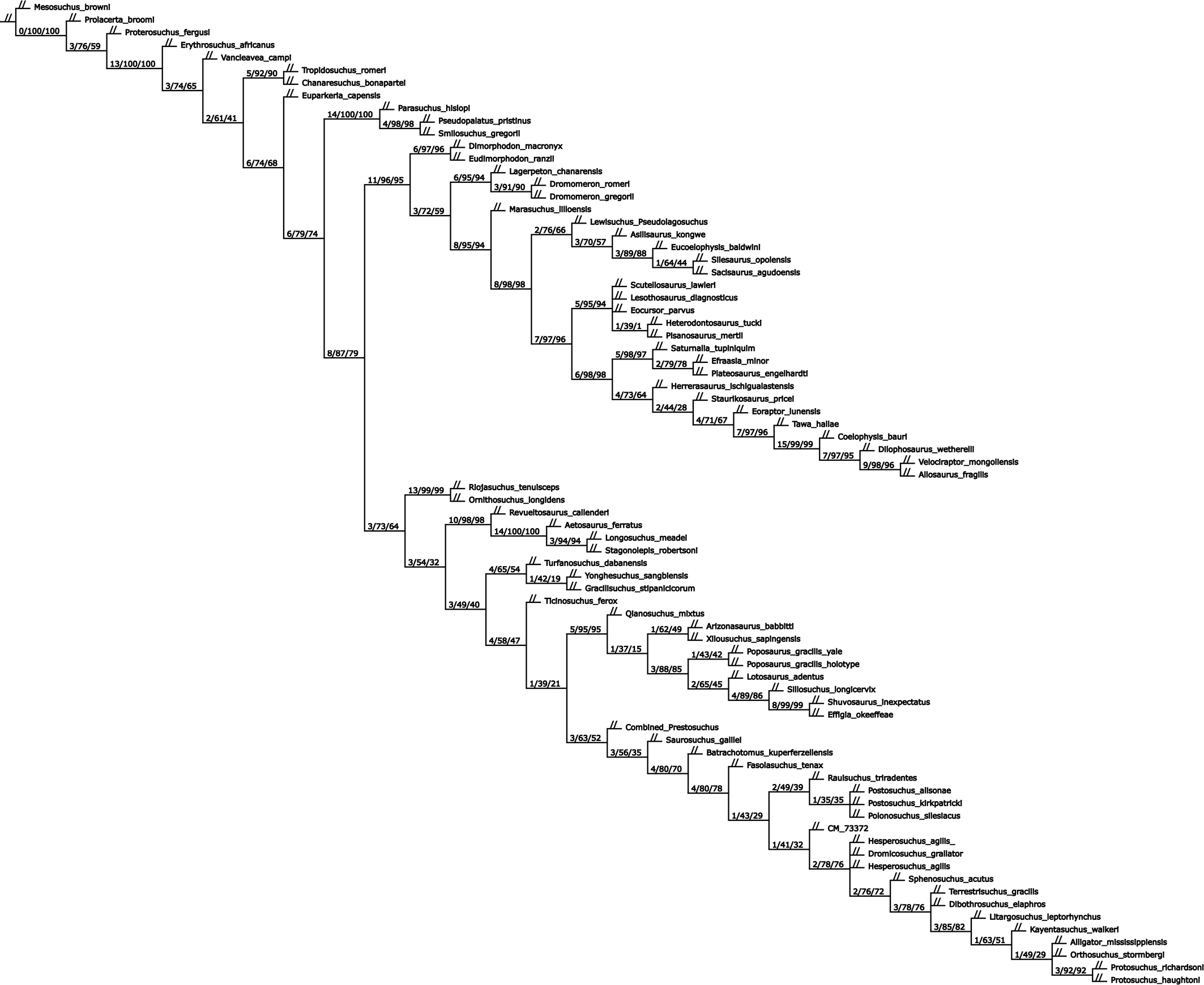

Supplement: Additional file 6 — Strict consensus tree for the main cladistic analysis when astragalus characters are all coded as uncertain for Turfanosuchus dabanensis , with nodal values for Bremer support and absolute and GC bootstrap frequencies. [file 1471-2148-14-128-S6.tiff]

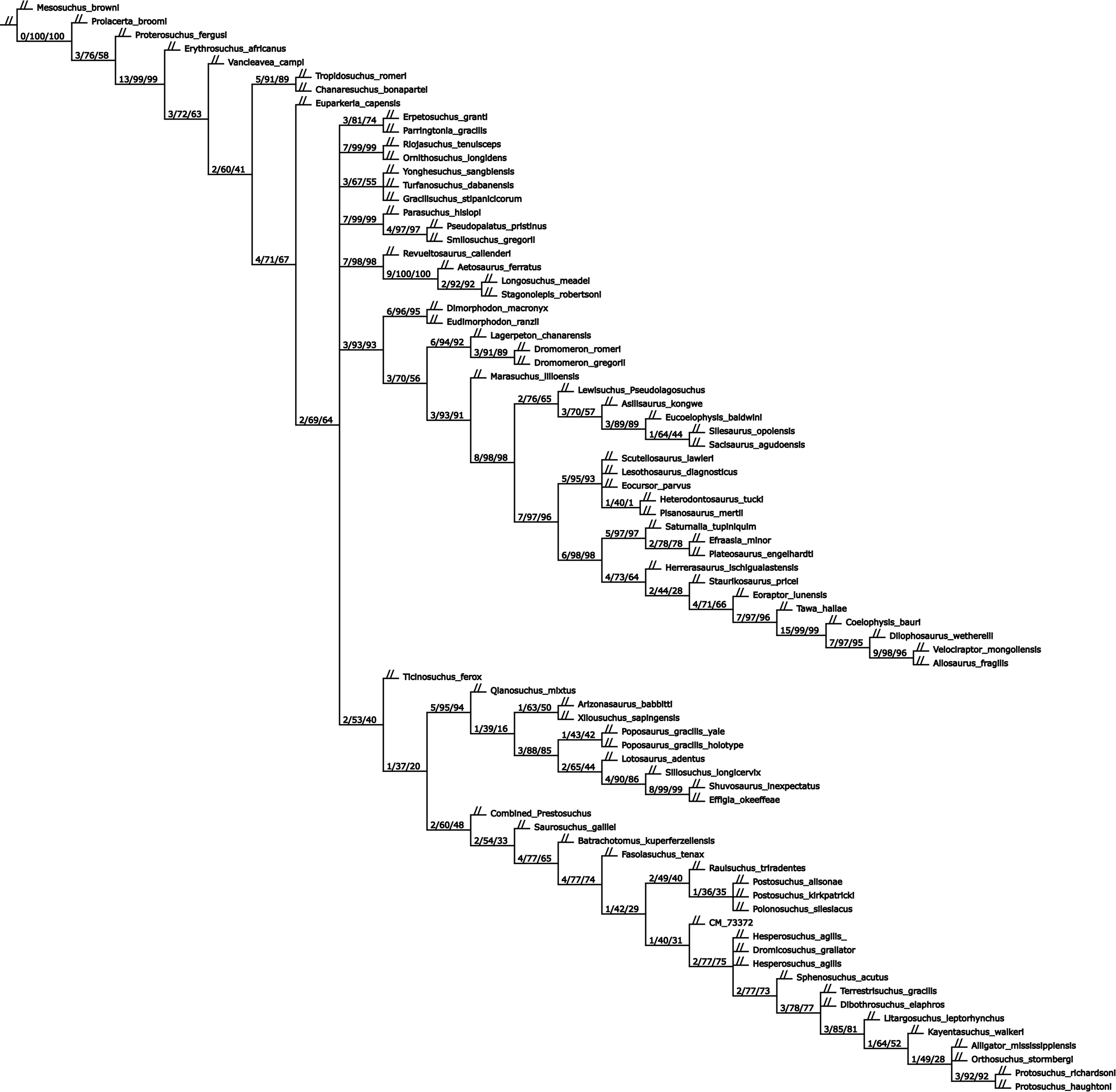

Supplement: Additional file 7 — Strict consensus tree for the cladistic analysis incorporating Erpetosuchidae when astragalus characters are all coded as uncertain for Turfanosuchus dabanensis , with nodal values for Bremer support and absolute and GC bootstrap frequencies. [file 1471-2148-14-128-S7.tiff]
